# Are Lovers Ever One? Reconstructing the Union Theory of Love

**DOI:** 10.1007/s11406-017-9860-x

**Published:** 2017-06-09

**Authors:** Elke Elisabeth Schmidt

**Affiliations:** Department of Philosophy, University of Siegen, Adolf-Reichwein-Straße 2, 57068 Siegen, Germany

**Keywords:** Love, Emotion, Union, Ontology, Definition, Cluster

## Abstract

Current analytical philosophies of romantic love tend to identify the essence of such love with one specific element, such as concern for the beloved person, valuing the beloved person or the union between the lovers. This paper will deal with different forms of the union theory of love which takes love to be the physical, psychic or ontological union of two persons. Prima facie, this theory might appear to be implausible because it has several contra-intuitive implications, and yet, I submit, it is more coherent and attractive than it seems to be. I shall distinguish three specific models and thereby offer a differentiated account of the union theory which has not previously been provided in the literature (1). I will claim that two of these models (the strong ontological model and the striving model) should be rejected (2). I shall then defend the third model (the moderate ontological model) against certain possible objections (3); but nevertheless, I shall conclude by showing how this model, too, faces further significant objections which ultimately expose the limits of the union theory of love (4). In conclusion, I will sketch the outlines of a non-reductive cluster theory of love.

From the time of Plato and Aristotle onwards, philosophy has offered quite different theories of love. Some of these can be interpreted as “reductionist” in character (Helm 2009, 52); they tend to identify the essence of love exclusively in one specific element in each case – whether it be in *concern* for a person, in the *valuing* of a person, or in a certain *union* of those who love one another. A further, perhaps non-reductionist theory of love is the *dialogue* model.^1^ In the following discussion I shall concentrate exclusively on contemporary forms of the so-called *union theory* of love that understands love as a sort of union, unity, fusion, or merging of two persons, as a “physical, psychological, or spiritual union between the lovers in which they form a new entity, the *we*” (Soble 1997, 66).^2^

The basic idea behind this theory can be traced back to the speech of Aristophanes in Plato’s *Symposium*. Irrespective of the controversial question as to how literally such mythical descriptions are to be taken in general, and regardless of Plato’s own attitude to such accounts, we may summarize Aristophanes’ mythical tale of “spherical” or “round” human beings as follows: in the earliest times, human beings were spherical in form, with each of them having four legs, four arms, and two faces. The Gods punished these beings because of their arrogance and thus chose to divide their bodies into two halves. This is, so the story goes, how two-legged humans first came into existence. But the resultant beings, each of whom had originally been part of a *single* (androgynous or mono-sexual) spherically formed human being, now yearned to find their other half to be reunited with it. To satisfy this striving of human beings for union, the Gods bestowed upon them the possibility of sexual intercourse and union. Hence, the sexual behavior of human beings and their capacity for love is explained on the basis of this desire to re-establish that lost state or condition of union.

In the context of contemporary philosophy, union theories of love are defended by only a few writers (theories of love in terms of “concern” are far more prominent) like Fisher (1990), Nozick (1989), and Solomon (1988). Writers such as Delaney (1996), Friedman (1998), Horn (2014), Scruton (2006), and Stump (2006) are found to have been significantly influenced by this theory.^3^ Although the union theory of love may offer some explanations for everyday expressions like “my better half,” it is just as likely to irritate many people since it seems quite implausible prima facie. We may very well ask, after all, what it could possibly mean to claim that the essence of love boils down to some psychological or even physical union. It seems particularly unclear how the entity comprising such union should be understood in philosophical and ontological terms and precisely how the individuality and autonomy of those who love each other relate to this union. One may, therefore, conclude that the talk of such union is merely to be understood in a metaphorical sense and is entirely incapable of yielding any deeper philosophical insight or meaning. Such talk of union would simply spring from the romantic notion of relationships involved in love, from specific forms of behavior and specific sentiments. Those who love one another would sometimes behave in ways that precisely *resemble* such union.^4^ By reducing the union theory in this way to merely a metaphorical expression, we effectively deprive it of any substantive meaning.

But this approach does not do justice to the union theory of love. Even without endorsing such a theory, we can still find it to be more plausible than it initially seems. In what follows, I shall distinguish between three specific models, thereby offering a differentiated account of the union theory which has not previously been provided in the literature (1). I will claim that two of these models (the strong ontological model and the striving model) should be rejected (2). Next, I shall defend the third model (the moderate ontological model) against certain possible objections (3). Nevertheless, I shall then show how this model, too, faces further significant objections that ultimately expose the limitations of the union theory of love (4). In conclusion, I will sketch the outlines of a non-reductive cluster theory of love.

## Three Models of the Union Theory of Love

Before proceeding to describe the three models of the union theory, we need to make three preliminary observations. In the *first* place, I shall be referring (at least for the most part) to cases of *romantic* love in terms of an initial general understanding of the issue – i.e., love between adult individuals which typically involves a sexual dimension along with the desire for reciprocity and an actively shared form of life. This does not always correspond precisely to the intentions of those who defend the union theory of love to the extent that while some of the latter indeed refer emphatically to romantic love (such as Nozick), others expressly wish their theories to be understood as theories regarding *all* kinds of possible forms of love and thus as a description that also includes the love existing between parents and children (see e.g. Fisher 1990). To avoid complicating the ensuing problems more than necessary, it seems rather reasonable to limit the discussion to romantic love (at least up to a certain point). In the *second* place, we shall only consider reciprocal love relations between *two* persons instead of poly-amorous relationships. This qualification is not intended to exclude the possibility that the union theory of love could be extended to cover love relations involving three or more persons (insofar as each party in such a relation loves every other party involved), even if this is generally excluded by the proponents of the union theory (cf. Nozick 1989, 82). In the *third* place, we shall concentrate on *reciprocal* instances of actual love relations.^5^ The question as to how the union theory of love relates to problematic and untypical cases of love relations (to cases of non-reciprocated love or to cases of love in relation to someone who is deceased) is certainly an interesting topic, but it is beyond the scope of this paper.

Let us now consider the various possible models of the union theory of love: the *strong ontological model*, the *striving model*, and the *moderate ontological model*.

1. *The strong ontological model* claims that the singular existences of two persons are absorbed in a new form of being when they love one another. This being is the union in question; both persons melt, as it were, into a single new entity. In this context, we may assume that (i) either the original individuals no longer exist as such, so as to completely dissolve in each other (as in the case of a chemical reaction where a new substance arises out of the combination of two others) or (ii) that the individual components continue in a certain sense and to a certain degree to exist within the context of this new union, as is the case with an organism such as the human body. While the latter does consist of the blood vessels and different organs, etc., which in their totality and their interaction constitute the body as a whole, they usually, considered on their own, cannot exist or can exist only in a very limited sense without the body as the entity that confers and sustains the union of their several functions. Here, too, the individual components only properly exist in their union with one another. Now the myth of the spherical beings, which we mentioned at the beginning, can be understood in terms of both these variants of the strong ontological model. Either way, the two original halves of a single spherical being strive to be reunified to become again what they once had been: *one*. In the philosophical literature on love, we occasionally find analyses which seem to fit this approach; some of them speak of shared or common forms of perception, while others talk about a complete relinquishment of autonomy on the part of the individuals involved (see below). In this connection, Fisher, for example, refers to “[f]used perception,” “[f]used emotion,” “[f]used action,“ and “fused deliberation and decisions” (Fisher 1990, 28).^6^ It is an undeniable disadvantage of this model in both of its variants that it is prima facie implausible: Those who love one another are still distinct individuals (and this already in an obviously corporeal sense); two persons do not really melt or fuse into one and both can continue to exist independent of their love. Therefore, this model is not convincing (and indeed is rarely defended in this rigid form).
2. *The striving model* of love posits no such total fusion between lovers and hence seems to enjoy greater initial plausibility compared to the previous model. According to this model, love is essentially understood as a *striving* for union, although this union cannot really be attained in principle.^7^ Therefore, union can be regarded, if at all, as realizable only to a certain degree; in any case, a complete union is not possible. – The striving model can certainly also be criticized. It may be objected that we should not understand love even as a striving for complete union (to the extent that this union is conceived in terms of the strict ontological model): for one thing, one should maybe not strive for something that is unattainable in the first place (because it is simply irrational wanting to have or be something one cannot get or be)^8^; for another, and more importantly, any such striving for a complete union would amount to the wish both of relinquishing one’s own autonomy and individuality as well as to the wish of abandoning one’s own self. But in case of love, as a rule, we do not strive (and indeed should not strive) to relinquish our own individuality and dissolve ourselves in the other (cf. Singer 1994, 24). What we strive for may well be a connection with the other which is as close and as mutual as possible, but it must preserve one’s own self as well.^9^
3. We thus come to the final model, namely the *moderate ontological model,* which attempts to take a middle way. According to this model, both individuals in one (for example, corporeal) respect continue to exist as distinct persons, while in another respect the very close connection – i.e., the love relation between them – does indeed give rise to a new and further entity, the union. This new entity is neither understood as simply metaphorical, nor is it just an object for which we strive; it is said to be something that actually arises. I shall present certain arguments to indicate the plausibility of this model in the following section.

## On Orchestras and Couples

How far then can we meaningfully speak of “union” in the context of love relations? It is easy to see in principle that the concept of union (or fusion) is indispensable in many contexts: In some cases, we may encounter unions in the strong ontological sense (one only has to think of the fusion of oxygen and hydrogen) in everyday experience, while we may face unions (unities) in the moderate ontological sense (I shall say more about these below) in other cases. Most objects consist of smaller parts and are what they are only in and through this connection of parts (molecules, trees, artificial objects like furniture); it is a commonplace that the whole is often more than the sum of its parts. In many contexts, it is undeniable that even human beings form unions (in the moderate ontological sense) that only exist *as* unions and can be understood in this respect as new entities. – In what follows I shall try to clarify this point in order to show why the talk of union is by no means more implausible in the context of love than in those other contexts.

For our purpose, “union” in the moderate ontological sense can be understood as *a connection of two or more entities (in this case: of persons) where such a connection is more than the mere sum of the entities concerned*. In this regard, the original entities continue to exist as individual beings, but together they can be said to fuse or to form a new entity. This can readily be seen if we think of a football team, a society,^10^ or an orchestra.^11^ An orchestra, for instance, can be understood as the union of a specific number of human beings who, taken together, are more than the mere sum of the parts: They form precisely the orchestra in and through which they act in common; their union is perceived as such by others, while the orchestra also understands itself as a union in a certain way. The orchestra is undoubtedly more than the sum of the musicians involved; the musicians also undoubtedly exist as distinct individuals even after joining the orchestra and similarly they can in principle exist as individuals even if the orchestra itself is disbanded.^12^

We can now transfer this basic thought to love relations between people. In a love relation there is a connection between two entities (persons) – it is more than the mere sum of the two persons and represents something new in the world.^13^ This connection is characterized by the fact that the parties involved enter into the closest possible relationship that is *much* more than a mere sum. Here both persons pay great attention to each other’s needs; they maintain shared preferences and interests; they value and respect one another; they take shared decisions; they are open to one another and trust one another; they pursue common ends; they share a sexual life; and they react to one another in various ways (also in relation to others). Theorists of love as union can appeal in this regard to *joint action theories*, which argue that two or more actors can perform *shared* acts of one kind or another on the basis of specific intentional attitudes. One variant of this theory claims that “it is the collective constituted by individuals rather than the individuals [...] which is the bearer of the collective intention” (Schmid and Schweikard 2009, 47); some writers even speak of “plural subjects” in this regard (Gilbert 2009, 168).^14^ Furthermore, one can perceive the couple in love precisely as a couple, one can behave toward them as a couple, and so on. And the intense connection between those who love each other can also be felt by themselves; literature and movies are full of scenes that express the feeling of fusion arising from the sexual act or in exemplary cases of concern for the other individual. Moreover, insofar as those who love one another – this is a central point – define themselves at least in part in terms of the other and partially form their own identities in interaction with the other, we may speak of an “entwined identity” (Horn 2014, 97) or “of the lover’s somehow identifying with the beloved” (Helm 2009, 39).^15^ But since identifying oneself with something does not mean *being* that thing in a full sense (one can also identify with a model or ideal), there is no need for us here to assume “any fusion in terms of hybrid ‘identities’” (Horn 2014, 97). The individual persons who become part of one couple may change (to a greater or lesser degree) through their relation to, and their interaction with, one another, but they also continue to exist as individuals. Hence, according to the moderate ontological model, we can say this: those who love each other continue to exist in their singularity, yet by the virtue of their connection – i.e., because of their love – they come to form a further real entity, a union, or, as Nozick would say, a *we*.^16^

Thus, what we have seen so far is this: two of the three presented models have proved to be less than intelligible. The strong ontological model proceeds on the basis of assumptions that no philosopher can really endorse; the striving model appears more plausible, but still brings problems of its own. The most promising candidate for a convincing theory thus remains the moderate ontological model.^17^ And such a model is indeed defended in the relevant literature: Nozick, for example, understands romantic love in the sense of this moderate theory of union. According to him, those who love each other become part of a “*we*” (“it feels to the two people that they have united to form and constitute a new entity in the world, what might be called a *we*,” Nozick 1989, 70). He goes on: “To be part of a *we* involves having a new identity, an additional one. This does not mean that you no longer have any individual identity or that your sole identity is a part of the *we*. However, the individual identity you did have will become altered” (Nozick 1989, 71 f.). And again: “In a *we*, the two people are not bound physically like Siamese twins; they can be in distant places, feel differently about things, carry on different occupations” (Nozick 1989, 70).^18^ Friedman understands love as a “*flexible* interpersonal equivalent to a federation of states” (1998, 166). In case of romantic love, he speaks explicitly of three entities: “In romantic merger, there is not merely one self, nor are there merely the two original selves; there are now three entities in a dynamic shifting interplay of subjectivity, agency, and objectivity” (Friedman 1998, 166).^19^ Fisher’s theory of union, on the other hand, must be interpreted in terms of the striving model of love. His concept of “fusion” proceeds on the assumption that the “fusion” in question can never be total: “[T]he process of fusion can never be completed, since the completion of the process would involve the lovers becoming one person” (Fisher 1990, 30; see also 27, and 44).^20^ Solomon, finally, speaks of a “fusion of two-into-one” (Solomon 1988, 26), but still emphasizes the unattainability in principle of any complete “fusion” (cf. Solomon 1988, 65, 68, 251), which again suggests an interpretation in terms of the striving model. At the same time, Solomon stresses the central role of the individuality of those who love each other while speaking of the “paradox of love” on account of the resulting dialectic (Solomon 1988, 65). His model revolves around the previously mentioned idea that the self is generally formed in a reciprocal relation with others and especially through the interaction with loving partners.

## Objections

Various objections are commonly raised against the union theory of love, some of which have already been touched on. I would now like to present them again in a concentrated form. The first three objections (a–c) can be disposed of relatively quickly; the last two objections (d–e), however, require more careful consideration. Let us start with the first three objections (a–c), which I shall try to meet with a single argument.

- *(a)* A bundle of objections whose aspects overlap one another relates directly to the prima facie implausible assumption of two persons somehow fusing into a *single* person. These objections partly focus on the ontologically questionable theses and in part simply on the physically and psychologically problematic implications involved. According to this argumentative strategy, two persons, even in a love relationship, would always remain two organisms that can reside in or occupy different places, and both persons would still be aware of themselves as such and would not take themselves for the other person in each case; while they might perhaps effectively perceive things simultaneously, their perception would still function separately (cf. Krebs 2015, 49; Soble 1997, 69). Similar objections draw attention to the problematic consequences from a logical point of view: two persons, as it has been alleged, could no longer interact with each other insofar as they are actually *one*.^21^
- *(b)* A further objection points out the incompatibility between union and autonomy; it regards the loss of autonomy as problematic or even as dangerous, since the assumption of a union is allegedly bound up with anti-individualist tendencies and implies a conflation of love with self-negation. Anyone who loves in this (strong union-theoretical) way would no longer be able to respect the autonomy of the other. Nevertheless, the respect for and encouragement of the autonomy of the other, critics claim, must be recognized as central in love.^22^ And even a critical feminist perspective finds purchase here insofar as it would regard the notion of love as self-relinquishment as a view that has largely been associated with the feminine as a result of traditional patriarchal structures (cf. Soble 2008, second edition, 159 f.; cf. Friedman 1998, 172–175).
- *(c)* Another objection, which has been raised with force by Soble, relates to the incompatibility of union and genuine (or robust) concern. In case of love, according to Soble, it must be possible to be concerned about the other for *his or her own sake*; but according to the union theory, one is only really concerned about *oneself* insofar as the object of love also belongs to this same self.^23^

These three objections do not need to be answered in detail because they all respond to the strong ontological model, which in both its variants affirms the *complete* fusion or union of those who love each other. But if we take the moderate ontological model as our point of departure, these objections no longer turn out to be compelling. For if we understand the union of individuals as an additional entity and continue to see those who love each other as individuals, this does not require us to accept any absurd assumptions regarding any ontological, physical, psychological, or logical matter. Moreover, no complete loss of autonomy on the part of the lovers is postulated (though some partial loss is acknowledged), and genuine concern for the other continues to be possible.^24^ It is, thus, an easy matter to counter objections a–c.

But there are two problems (d–e) that the union theorist is unable to resolve simply by appealing to the moderate ontological model. Let us now look at these problems more closely.

- *(d)* If all love relations, according to a further objection, were indeed new entities, this would lead to a superfluous extension of the ontological constituents of the world. In accordance with the heuristic principle of parsimony, any additionally postulated entities could only fall victim to Occam’s razor. – We could respond to this objection as follows: The union theorist must undoubtedly concede that the number of assumed entities has certainly increased on the basis of relevant premises, but this is hardly an implication that needs to be avoided at all costs. If musicians come together to establish an orchestra, then something specifically new in the world emerges (the orchestra); if a boat is constructed out of nails and wooden planks, then a new entity, a “boat,” also arises; if atoms (or whatever precisely there may ultimately be) are combined to form a table, then there is henceforth such a thing as a table. Since there is no question that in all these cases we can say that new entities have actually been created, it also seems justified in case of love to assume new entities without violating the principle of parsimony. If, however, the advocate of the principle of parsimony would continue to criticize the assumption of further, allegedly unnecessary, entities at this point, one could still question the principle of parsimony itself.
- *(e)* Let us finally consider one further objection: two friends, one might object, also establish a connection (a relation of friendship) in which their original individual characteristics are partially preserved and that is more than the mere sum of the individual persons concerned. Friendship is by no means exhausted in the mere existence of the persons involved; therefore, so the objection goes, there is a new entity or union not merely in the case of romantic love but also in friendship.^25^ (And one may understand the relation between family members and possibly also other interpersonal constellations in a similar way). On this account, according to the present argument, a *multiplicity* of interpersonal relations gives rise to unions in the sense described and therefore the formation of a union would certainly not be a uniquely characteristic feature of romantic love. Moreover, and in direct connection with this point: If in the case of friendship or family relations people actually speak of unions, it would seem conceptually inappropriate to use this term, for the concept involved would basically seem reducible to “relation.” The concept of union, on this objection, would therefore be only subjected to an inflationary use and finally simply indicate the presence of (various kinds of) relationships. – This objection (namely that we also encounter unions in the case of friendships, etc.) is much more difficult to counter than the earlier ones. There are two options here: *Either* the union theorist has to bite the bullet – insofar as friendship is also a connection between two persons which is more than the mere sum of both, we can speak of union here as well (and this would be the end of the story) – *or* the union theorist must specify the concept of union at this point: while it is quite true that the aforementioned cases (friendship etc.) involve connections between two persons which are more than the sum of the parties in question, these connections can be of a stronger or a weaker nature; some friends may share a relatively short period or domain of their life in relation to one another, whereas those who romantically love each other may as a rule share much more with one another. Now the union theorist might try to locate unions at the higher end of this scale where romantic love is to be located and a special depth and intensity constitutes the connection between two human beings. In this regard, we could well speak in all other cases of new entities in the moderate ontological sense, but not indeed specifically of *unions*.^26^ However, this seems rather arbitrary: Why should we not speak of union in cases of very close friendship, too? (And would the piece of furniture no longer be a union in this sense?) However, the union theorist would be able to answer as follows: Romantic love, he could say, is characterized by the fact that it presents us with particularly *deep* forms of union that are of a particular kind and have specific features. Thus, the presence of such a *deep* union could indeed be understood as a sufficient feature or, more precisely, as the *definiens* of romantic love (thus we should speak, more precisely, of the *deep union theory* of romantic love).^27^

So far so good. Let us sum up: We have seen that the first three objections (a–c), which can initially be raised against the union theory of romantic love, are easily countered by the moderate ontological model. The union theory is also capable of responding to objections d–e, at least up to this point of the argument. However, there are still some further questions to answer here.

## What Remains to be Said for the Union Theory of Love?

Is it still possible, then, to maintain the union theory of romantic love? If we do wish to defend a union theory of love, it looks as if the moderate ontological model would represent the only promising candidate as far as a philosophically reliable position is concerned. Nonetheless, and here we come to the decisive point, it turns out that “romantic love” can neither be *defined* by the presence of a union in the moderate ontological sense, nor can the presence of such a union be understood merely as a *sufficient* condition of such love. One could, as we have just seen, respond to objection (e) by regarding the presence of particularly *deep* or intensive forms of union as sufficient for the presence of romantic love. But it is by no means obvious why we should not also be prepared to recognize the presence of a similarly deep union in the moderate ontological sense in the early love between mother and child, for instance. We might point out, with Fisher, that the mother also experiences the infant – at least in some cases – as part of herself, while the infant is not yet in a position to distinguish between the mother, the world, and itself; the life of the mother may completely revolve around the needs of the newborn, while the child may exist by completely depending on the mother.^28^ Another example of a deep union that is not romantic love is often claimed to be found in the mystical love to God; and maybe the deep, but not romantic, love between twins (siblings) is another example. But if deep unions are also possible in other cases, the presence of such a union cannot possibly be a sufficient and distinctive feature of romantic love. And that can only mean that the essence of romantic love does not consist in the presence of a deep union after all. Rather, the presence of such a deep union is only (if at all) one feature amongst several such features of (fully realized) romantic love.

Here it would still be possible for the union theorist to develop a concept of a deep and a specifically *romantic* form of union that would include, for example, the aspects of sexuality, concern, trust, and the creation of a shared life. On this account, the (deep) union would basically be the *genus proximum* for a definition of romantic love; the *differentia specifica* of romantic union could then be located in the particular aspects we have just mentioned. Yet the ascription of additional characteristics, such as sexuality or concern (i.e., the ascription of the *differentia specifica*), is highly problematic: There are such things as asexual love, or jealous love marked by distrust, or loving relationships of people who do not, for whatever reason, wish to share their life in the long term, and so on. The notion of “concern” (or “care”) may indeed look like a promising candidate to specify the definition, but there is no doubt that this is just as much a central feature of friendship and of the love between parents and children. It cannot therefore be adduced (even in combination with the presence of union) as an unambiguous distinguishing characteristic here.^29^ And it turns out to be the same in every case: Whenever specific and apparently necessary elements of a romantic notion of union are proposed, it is always possible to think of counter examples that challenge the necessity of these elements. Moreover, it is clearly not the actual presence of particular elements (desire, concern, etc.) which is required, rather it is the concept of disposition that must be brought into play here in one way or another (for those who love each other are not *always* concerned about one another). And we should also take account of the temporal aspects involved here, such as the question as to how we would think about a couple who once enjoyed sexual intimacy but from a certain age or point of time have ceased to do so.

We must acknowledge, therefore, that the presence of union in the moderate ontological sense is not a sufficient criterion for the presence of romantic love. At best, the presence of such union could be understood as a necessary element of love in general, including the love between parents and children and other forms of love (insofar as we are ignoring problematic cases here).^30^ Hence, however much may be said to make the union theory of romantic love appear plausible (and it is certainly not absurd), it cannot, in the final analysis, be regarded as satisfactory.

In the light of the difficulties we have mentioned, however, the attempt to discover *any* definition of romantic love seems equally hopeless. If we look for several necessary features (such as concern, sexuality, specific emotional-phenomenal qualities, etc.), which, taken together, could seem sufficient to identify cases of romantic love (whether union is understood as one of these necessary features or not), we can always find counter examples that challenge the necessity of these features. Now one might object that such problematic examples represent extreme rather than paradigmatic cases and that we could at least still manage to define an *ideal* or *typical* love relation (especially since definitions are always somehow normative in character). Yet, once again, this seems hardly promising or even desirable because why should we think it justifiable, for example, to rank asexual love as non-ideal and thus as inferior? The fact that such love is not typical – i.e., that it does not correspond to the average case – does not show that it is in any normative sense deficient.

This presents us with a fundamental problem: A definition furnishes the essence of something and answers the question “what is x?” Should the fact that no convincing definition of love can be found lead us to conclude that love has no essential character at all? Must we perhaps content ourselves simply by identifying certain family resemblances here? But this does not seem very plausible either. For we are all familiar with love; we usually recognize love when we encounter it and sometimes we think that someone is merely imagining it. We believe that we know what love is and in any case that it certainly does have an essential character. And this familiarity with the phenomenon suggests one final possibility: Perhaps romantic love could be defined through a (partially) variable cluster of properties (although this would naturally mean abandoning the classical theory of definition).^31^ According to this model, we could draw from a pool of, let us say, 15 conditions – *none* of which is either *necessary* or *sufficient* – and say, for example, that seven of these conditions must be present in any case of romantic love. Precisely *which* seven out of the 15 are relevant in a particular case would not be given in advance, but the presence of a certain union could be an element in this pool. We could thus list the characteristic 15 features *a* to *o* (union, concern, trust, affection, shared feelings, desire, jealousy, attractiveness, the wish to spend time together, permanence, etc.), and if at least seven elements from this set, whatever they may be, perhaps *a* to *g* or *i* to *o*, are present, one would be justified in speaking of romantic love. In this sense, there could be cases of love which exhibit no common properties, apart from precisely possessing seven (different) features from the given pool. But then there could also be love without union. This seems to me to be the most promising model, although it certainly remains unclear how large the pool of potential characteristic features of love would really need to be, precisely how many characteristic features must be present in order to be able to speak of romantic love, and which characteristic features belong to this pool at all. Perhaps one could postulate some necessary core elements in this pool (such as union, for example) that must be present in every case. Still, a particular number of further variable characteristic features from the pool must gather around those core elements if we are to speak of romantic love in a concrete case. And then we could say: It cannot be that two people love one another if they have never created a certain union, although it alone is not sufficient. To explore all this would be part of a larger project.

## Footnotes

1 For a defense of a theory of love as “concern” or “care,” see Frankfurt (2004); for a theory of love in terms of valuing, see Velleman (1999); and for the dialogue model, see Krebs (2015). – I should like to thank an anonymous reviewer, Aaron Ben-Ze’ev, Susanne Schmetkamp, and Dieter Schönecker for making some very valuable observations and objections, and Nicholas Walker for translating the text into English (with the exception of Hegel, all German sources are translated into English by N.W.).

2 The *Historisches Wӧrterbuch der Philosophie* describes love in a rather comparable way as the general concept for “relations which establish unity” (Schöpf 1980, 290).

3 Theories of love conceived in terms of union are also particularly well represented in theological contexts, although I shall not be able to discuss all these approaches here. I shall also have to refrain from discussing sociological or psychoanalytical contributions in the present context.

4 Solomon (1988, 66 f.) refers to a similar reading. One variant of the metaphorical reading regards the mere *feeling* of union as essential to love, while the corresponding union does not actually have to be present. One might think that this is how the following remarks of Nozick could be understood best: “The heart of the love relationship is how the lovers view it from the inside, how they feel about their partner and about themselves within it [...]” (Nozick 1989, 74). Yet, this interpretation of Nozick’s theory will turn out to be misleading.

5 The sexual orientation plays no particular role here.

6 There are certain passages in Hegel’s work which are occasionally interpreted as examples of the strong ontological model of the union theory of love. Hegel writes: “But its objective source [i.e., of the institution of marriage] lies in the free consent of the persons, especially in their consent to make themselves *one person*, to renounce their natural and individual personality to this unity of one with the other. From this point of view, their union is a self-restriction, but it is in fact their liberation because in it they attain their substantial self-consciousness” (Hegel 1821, § 162).

7 Nozick also sometimes emphasizes the *striving* for union: “Love, romantic love, is *wanting* to form a *we*” (1989, 70). But in the course of his text he goes on to describe a situation where a “*we*” is actually present.

8 Though one could argue that we may strive to be perfect in many domains, knowing that perfection is unattainable. – I am grateful to the reviewer for this valuable objection.

9 Singer argues that the striving for union only leads to a situation where “men and women distort themselves in one respect or another” (Singer 2009, 19; cf. Singer 1994, 29).

10 Consider the passage from *The Social Contract* (1762) where Rousseau speaks of “the total alienation of each associate, together with all his rights, to the whole community” (Book I, Ch. 6). A little further on we read the following: “At once, in place of the individual personality of each contracting party, this act of association creates a corporate and collective body, composed of as many members as the assembly contains voters, and receiving from this act its unity, its common identity, its life, and its will.”

11 The examples we have cited and the corresponding questions they raise (also) fall within the field of *mereology*. It will not be possible to enter here into the relevant debates (and the issues, of course, are discussed controversially).

12 One might, of course, object that musicians as orchestral musicians are different from musicians who are not involved in an orchestra anymore or that their characteristics (or roles) can change when they actually leave an orchestra. And one could object, on account of such changed characteristics, that they are no longer precisely the *same* persons, just as an old man is no longer the same person as the former schoolboy. Similarly, we could go on to pursue fundamental reflections on the (non-)constancy of personality itself. In the present context, however, I prefer to proceed on the basis of the idea that there is a constant core to personality (however limited that may turn out to be).

13 For the objection that this usage of “union” (a connection of two persons where this connection is more than the mere sum of its parts) is quite another as that of “union” in the context of love, see below objection (e). – I am grateful to Aaron Ben-Ze’ev for raising this objection.

14 Claims to the contrary are specifically described as “reductionist” in character (Schmid and Schweikard 2009, 53).

15 Perhaps this is what Nozick means when he claims that “[e]ach becomes psychologically part of the other’s identity” (Nozick 1989, 72).

16 What I described above as central elements of a union (interacting with one another, helping and trusting each other, sharing interests, values, feelings, decisions and ends, being open to one another and doing things together, and so on) are also central aspects of the dialogue model of love. Nevertheless, the focus of the union model is different from the focus of the dialogue model: While the dialogue model emphasizes the interaction and sharing between lovers, the union theory underlines the fusion of lovers in a more ontological sense. Maybe one could argue that according to the union theory, the union *supervenes* on those elements (sharing, interaction, etc.), but is not reducible to them.

17 As an opponent of the union theory of love, Singer (1994) points at various common examples of “merging” processes, as in the case of salt (the “conjunction of sodium and chloride,” 18), of the United States (20) or of sperms and egg cells (26). But he regards the fusion of two persons as something quite impossible. His criticism, however, only makes sense in relation to the strong ontological model.

18 It is not altogether clear which view Nozick adopts regarding the question as to whether we are really dealing with an actual new entity in an ontological sense in the case of love. He responds to this issue as follows: “This resembles the question of whether a whole society is an additional entity in the world or merely the sum of the web of various people’s relationships. Is a human body an additional entity in the world or simply those constituent physical parts in a web of relationships? Like a body or a society, a *we* maintains itself and adapts in the face of (a wide range of) new circumstances [...] Noticing the multifarious features of the *we* and the new activities and value it makes possible is more important than deciding whether it constitutes a new item of ontological furniture in the world” (Nozick 1989, 73, footnote). Yet, his further observations seem to suggest the moderate ontological model. It is quite clear, however, that the strong ontological model is definitely excluded.

19 See also Delaney (1996, 341). According to Friedman (1998, 163), the emergence of such a union is by no means restricted to the realm of love.

20 Nonetheless, even in Fisher’s and Nozick’s texts it is possible to find certain passages that, read on their own, would seem to suggest the strong ontological model (see, for example, Fisher 1990, 28: “[W]e come to perceive, feel and act as a single person, so that the perception, feeling or act does not exist unless both persons participate in it, and neither can say which of the two has originated it”). But such passages must be taken strictly in context.

21 See, for example, Soble (1997, 72, footnote); Krebs also points out the danger of a “standstill” if union is conceived in this manner (Krebs 2015, 50).

22 See, for example, Krebs (2015, 50), Helm (2005), and Singer (1994, 30, 135–139). Friedman points out that the relinquishment of autonomy can take place in an unequal manner (1998, 169–172), whereas a temporary loss of autonomy can mean a long-term gain in autonomy (1998, 176).

23 Cf. Soble (1997): “[...] for in union-love there are not two persons genuinely concerned about each other but only one enlarged person caring for itself” (71). Delaney (1996, 341 f.) points towards a rather similar direction, albeit in a somewhat less critical way.

24 Friedman argues the same way (1998, 166). Scruton speaks of a “‘community of interests’ that erodes the distinction between *my* interests and *his*” (Scruton 2006, 231; cf. Hunter 1980, 74 f.) and may to some degree seem vulnerable to Soble’s objection. Yet, he goes on to add: “I can dissociate myself from some of your desires, and to some extent. But that is only because there are degrees of love” (Scruton 2006, 230).

25 Aristotle’s (broad) conception of friendship also establishes a connection between friendship and union in a specific sense: The well-being of one friend is bound up with that of the other and vice versa. On this, see Sherman (1993), who speaks of an “extended [self]” (94) regarding Aristotle’s understanding of friendship and claims that “one’s happiness becomes extended to include the happiness of others [that is: of friends]” (101). But it remains quite clear for Aristotle that “within this extended and interwoven life, the individuals nonetheless retain their separateness” (103).

26 Stump, for example, appears to see the matter rather differently when she speaks of “different kind[s] of union” with a person which are “appropriate for the people who are his spouse, parent, child, colleague, and priest [...]” (Stump 2006, 31).

27 When I claim in what follows that specific features are “sufficient” for love, I mean that they can serve as a definition of “love.”

28 Fisher also defends the union theory in relation to all forms of love and therefore also to the love between parents and children. According to him, the greatest degree of fusion obtains at the beginning of life, namely in the context of the relationship between mother and child; he claims that this union decreases as the child grows and matures, and that the child gradually separates itself from the mother (cf. Fisher 1990, 39 and 70 ff.). – In contrast to Fisher, one could argue that to have an autonomous self is a necessary condition for building a union and that an infant does not have such an autonomous self and hence cannot build a union with its mother. It seems to me, however, that in case of the union of a mother and her child, there is at least the mother’s autonomy which is sufficient to build a union regardless of the question whether the other self is autonomous or not.

29 One should add in passing that, in some sense, love as union and love as care or concern are closely connected with one another. Nozick’s *general* model of love (1989, 68 f.), for instance, can also be understood in a certain sense as a model of concern (insofar as the well-being of one party is bound up with the well-being of the other), although for Nozick union constitutes the specific characteristic of romantic love. According to Fisher, love involves two essential elements, namely “the development of a fused self” (1990, 26) and what he calls “humble benevolence” (actively caring for the other in accordance with the thoughts and feelings of the other, cf. 20 and 96). For Fisher, the union (“fusion”) arises from the concern in question (1990, 27). According to Delaney, concern is an aspect of the desire for union (cf. Delaney 1996, 340). Here the basic thought must be that *being one* with the other means sharing my interests with the other and making the interests of the other my own, or vice versa: If I make the interests of the other my own, it increasingly leads me to adopt his or her standpoint and hence I *become* the person that I love. Stump (2006, 27) also understands love (following Thomas Aquinas) essentially as the interplay between the wish for the well-being of the one who is loved and the wish for union with that person (although in her case it looks as though concern and union are not directly connected). On the other hand, Frankfurt’s attempt to understand love (generally) as an obligating and disinterested volitional structure in the sense of care for another person does *not* confuse or conflate care with union (cf. Frankfurt 2004). For Scruton, concern and union are also connected insofar as he expressly speaks of a “union of interests” (2006, 229).

30 Such problematic cases would be those of unfulfilled or unreciprocated love or of love for those who have died. While in a certain sense these may not represent ideal-typical cases of love, they are obviously by no means rare instances of love. Human beings can love others even when they are not loved by these others in return; people can also love their recently deceased partners. But in such cases as these how can we still meaningfully speak of the presence of particular characteristic features such as union?

31 We find a proposal of this kind in Newton-Smith (1989, 204 f.).

## References

[CR1] Delaney N (1996). Romantic love and loving commitment: Articulating a modern ideal. American Philosophical Quarterly.

[CR2] Fisher M (1990). Personal Love.

[CR3] Frankfurt H (2004). The reasons of love.

[CR4] Friedman M (1998). Romantic love and personal autonomy.

[CR5] Gilbert M, Schmidt HB, Schweikard DP (2009). Zusammen spazieren gehen: Ein paradigmatisches soziales Phänomen. *Kollektive Intentionalität. Eine Debatte über die Grundlagen des Sozialen*.

[CR6] Hegel, G. W. F. (1821). *Elements of the philosophy of right*. Translated by M. Knox and edited by St. Houlgate. Oxford: Oxford University Press 2008.

[CR7] Helm, B. W. (2005). Love. *Stanford encyclopedia of philosophy*. https://plato.stanford.edu/entries/love/. Accessed 12 Jan 2017.

[CR8] Helm BW (2009). Love, identification, and the emotions. American Philosophical Quarterly.

[CR9] Horn C, Mertens K, Müller J (2014). Liebe als soziales Phänomen: Intersubjektivitätstheorien. Die Dimension des Sozialen. Neue philosophische Zugänge zu Fühlen, Wollen und Handeln.

[CR10] Hunter JFM (1980). Thinking about sex and love.

[CR11] Krebs A (2015). Zwischen Ich und Du. Eine dialogische Philosophie der Liebe.

[CR12] Newton-Smith W, Soble A (1989). A conceptual investigation of love. *Eros, Agape and Philia. Readings in the Philosophy of Love*.

[CR13] Nozick, R. (1989). Love’s bond. In R. Nozick (Ed.), *The examined life. Philosophical meditations* (pp. 68–86). New York: Simon & Schuster.

[CR14] Rousseau, J.-J. (1762). *The social contract and discourses*. Translated by G. D. H. Cole, revised edition, London 1973.

[CR15] Schmid HB, Schweikard DP, Schmid HB, Schweikard DP (2009). Kollektive Intentionalität. Begriff, Geschichte, Probleme. Kollektive Intentionalität. Eine Debatte über die Grundlagen des Sozialen.

[CR16] Schöpf A, Ritter J (1980). Liebe. Historisches Wörterbuch der Philosophie.

[CR17] Scruton R (2006). Sexual desire. A philosophical investigation.

[CR18] Sherman N, Badhwar NK (1993). Aristotle on the shared life. Friendship: A philosophical reader.

[CR19] Singer I (1994). The pursuit of love.

[CR20] Singer I (2009). Philosophy of love. A partial summing-up.

[CR21] Soble A, Lamb RE (1997). Union, autonomy, and concern. Love analyzed.

[CR22] Soble A (2008). The philosophy of sex and love. An introduction.

[CR23] Solomon RC (1988). About love: Reinventing romance for our times.

[CR24] Stump E (2006). Love, by all accounts. Proceedings and Addresses of the American Philosophical Association.

[CR25] Velleman JD (1999). Love as a moral emotion. Ethics.

